# Sudden Death from Spontaneous Rupture of the Stomach

**Published:** 1845-11

**Authors:** 


*Sudden Death from Spontaneous Rupture of the Stomach.* By Sig. Morici.—A man, 30 years of age, who had been several times cured of intermittent fever, was seized again with the same disease on the 21st of January. Quinine and other remedies produced a cure in ten days. A bitter decoction, however, was continued for five days longer, during which he had no fever, when on the fifth day he was suddenly seized with violent pain in the lumbar region. There was, however, no fever, no swelling, redness or abnormal heat, nor hardness at the seat of the pain. The pain was aggravated on pressure, and the patient continually sat erect in bed, as it was impossible for him to lie on his back or side. Next day feverish symptoms made their appearance, accompanied with retraction of the right testicle, difficulty of making water, and a sensation of constriction in the sphincter of the anus. By the third day the fever had abated, and he was even able to take a little soup. In the evening he rose to go to the water closet, and in returning to his bed fell down dead.

The thoracic viscera were found healthy, as were also the abdominal, with the exception of the stomach, which was ruptured on its anterior surface, almost in its very centre. The aperture was about three fingers' breadth in length, and had allowed the contents to escape among the abdominal viscera. Although the margins of the aperture preserved their natural texture, they were slightly hypertrophied and dotted with redness. The same dotted redness was remarked on the mucous membrane of the stomach a short distance around the rupture, but the membrane retained its natural texture, and had undergone no pathological changes.

Sig. Morici could not account for the production of the rupture, unless it were the straining at stool acting on a part of the stomach, weakened by circumscribed inflammatory action of the mucous membrane, as indicated after death by the dotted redness. In this, as in all other recorded cases where the stomach has been found ruptured, the death was sudden, and was not attended with hæmorrhage, or rupture of any arteries or veins of importance.—*Ibid, from An. Univ. di Med.*

